# Analysis of the Tensile Properties of Composite Material Added Carbonisate Based on the Change of Strain Dynamics

**DOI:** 10.3390/ma17246219

**Published:** 2024-12-19

**Authors:** Agata Wieczorska, Grzegorz Hajdukiewicz

**Affiliations:** Faculty of Marine Engineering, Gdynia Maritime University, Morska St. 81-87, 81-225 Gdynia, Poland; g.hajdukiewicz@wm.umg.edu.pl

**Keywords:** Kolmogorov-Sinai metric entropy, carbonisate, pyrolysis, composite tensile strength

## Abstract

This paper presents the application of Kolmogorov–Sinai ($E_{K-S}$) metric entropy calculations performed on experimental data sets (relative elongations ε) recorded during static tensile testing of a composite material with carbonisate. The $E_{K-S}$ calculation method makes it possible to represent the dynamics of strain change occurring during the endurance test. The depiction of the change in the dynamics of elongation compared to the course of the tensile curve makes it possible to analyse the strength properties of the tested specimens. The material used for the study is a layered epoxy composite with the addition of carbonisate obtained by pyrolysis from organic waste from MDF (Medium Density Fibreboard) furniture boards. For the tested material, two variants were prepared without the addition of carbonisate (samples designated as I and III) and two variants differing in the percentage of carbonisate: 5% by mass (sample IV) and 7.5% by mass (sample II), with a constant fraction of 0.5 mm. Analyses showed a slight deterioration in the tensile properties of composites containing carbonisate. SEM (Scanning Electron Microscope) studies of the carbonisate samples revealed the presence of cracks, pores and local delamination, which correlates with a reduction in strength parameters. For sample II, the tensile strength (R_m_) was 9.032% lower compared to the base sample I and the tensile strain decreased by 0.098%. For sample IV, a decrease in parameters was also observed compared to base sample III—the strength decreased by 13.29%, and the tensile strain decreased by 10.64%. The results obtained in this study were additionally decided to be analysed using metric entropy calculations, which makes it possible to capture significant qualitative changes occurring in the structure of the tested samples not depending on the results of the static tensile test. In the context of epoxy composites with the addition of carbonisate, this analysis can contribute to a better understanding of the influence of the carbonisate obtained in the pyrolysis process on the structure of the composite and its performance properties.

## 1. Introduction

The modern development of composite materials represents a significant step toward optimising their physical, mechanical and thermal properties, which are widely used in many industries such as automotive, aerospace and construction. Among the various polymer matrices, epoxy composites are gaining special attention for their excellent adhesive properties, high mechanical strength and chemical resistance. However, one of the challenges associated with their production is the need to improve selected properties, such as thermal resistance or wear resistance. In this context, the introduction of various additives, such as nanomaterials or thermal process products, into the polymer matrix is becoming increasingly important [1,2].

One such promising additive is carbonisate, obtained by the pyrolysis of organic waste. Carbonisate, as a carbon-rich product, has interesting properties that can significantly affect the structure and properties of epoxy composites. The pyrolysis process, which involves thermal degradation of the material under anaerobic conditions, yields products with high carbon content that can be an attractive reinforcing additive in polymer matrices. The introduction of carbonisate into epoxy composites can lead to improvements in their mechanical and thermal properties, as well as resistance to environmental factors.

The paper [3] focused on analysing the effect of graphene nanoplatelets (GnP) on the mechanical properties of epoxy plates with initial edge cracking. Special attention was paid to the first mode of the normalised stress intensity factor (SIF), a key parameter characterising fracture toughness. Comparison of the results obtained by the Bayesian method with analytical solutions provided new data on the accuracy and efficiency of the test methods used. In the work [4], polymer–wood–epoxy composites (WEPC) were developed using epoxy monomers introduced into the cell cavities of plantation wood. The resulting materials exhibited excellent mechanical properties and optimisation of the stress–strain system allowed for increased resistance to mechanical loads. Research [5] focused on laminated epoxy nanocomposites reinforced with graphene nanoplatelets at low levels of this additive. FEM analysis and classical lamination theory (CLT) were carried out, investigating the effect of the quasi-isotropic, cross and unidirectional laminate orientation on the stress distribution across the laminate thickness. The results showed that the appropriate choice of volume fraction and additive orientation in the material structure is crucial for optimising the mechanical properties of composites. The paper [6] describes the study of epoxy–glass composites with the addition of recycled rubber. The analysis of experimental results carried out with the use of Kolmogorov–Synai (*E_K-S_*) metric entropy calculations allowed a broader assessment of the strength properties of this material. These results showed that the use of recyclate significantly changes the mechanical properties of the composite while also bringing a new perspective to materials research through the use of statistical tools. On the other hand, the article [7] studied the effect of graphite content on the properties of polyester-glass composites with the addition of recyclate. The results showed that increasing the amount of graphite causes a decrease in the mechanical properties of the composites but is not directly proportional to the amount of additive. Materials made by hand lamination were subjected to tensile tests by standards, which made it possible to determine their mechanical characteristics in detail. A sustainable approach to the design of building materials is becoming increasingly important in the context of the global challenges of environmental protection, reduction of greenhouse gas emissions and a closed-loop economy. In this context, the development of innovative materials, such as composites enriched with carbonisate, may provide an alternative solution. Carbonisate, extracted through the pyrolysis process of MDF boards, is an example of a secondary material that can be used as a sustainable additive to a composite, allowing its environmentally friendly use. The pyrolysis process allows for efficient processing of MDF waste, which reduces the amount of landfill waste, while also fitting in with the principles of the circular economy. The use of carbonisate in building materials can help improve their durability, structural integrity and energy efficiency, as indicated in studies by Mahmoud Sodanga et al. [8] and Elisabetta Negro et al. [9]. However, to fully realise the potential of this material, detailed research and analysis of its effects on the strength and tribological properties of composites are needed. The above works provide valuable data on the effects of various additives and modifiers on the properties of epoxy and polyester–glass composites. A study [10] on the hardness of epoxy composites with the addition of carbonisate as filler showed that the use of this material leads to a significant increase in hardness compared to the reference sample A0 (without the addition of carbonisate). Statistical analysis confirmed that the addition of carboniser not only improves the mechanical properties but also provides greater repeatability of results. The data obtained provide a valuable basis for the further development of epoxy composites with improved parameters, opening up new possibilities for their use in industries requiring high-strength materials, such as the construction, automotive and aerospace industries.

To better understand how the newly formed material behaves under load, a statistical analysis method based on metric entropy was used. This approach allows changes in deformation dynamics to be tracked during testing. The use of the results of Kolmogorov–Synaya (K-S) metric entropy calculations in the analysis of the strength properties of materials is relatively novel [11,12]. Studies [11,12,13,14] have shown that with modern strength equipment, as well as the *E_K-S_* calculation and acoustic emission (AE) method, it is possible to precisely determine the transition point of a composite material from the elastic to the plastic phase. Analysis of the experimental data, based in this case on the values of strain ε, makes it possible to identify the changes occurring in the structure of the material, including its internal structure, under load. The premise of this method is to relate structural changes in the material to a specific measurement point, which determines the critical moment—the boundary between the elastic deformation phase and the elastic-plastic phase. The application of this technique in the study of epoxy-glass composites with the addition of carbonisate after the pyrolysis process of MDF furniture boards makes it possible to determine the stress values within which the material can be operated safely.

This thesis focuses on the analysis of the effect of the addition of carbonisate, obtained by pyrolysis, on the strength properties of epoxy composites, using the *E_K-S_* calculation method. The research aims to evaluate the potential benefits of this additive and its effect on changing the mechanical properties of the composites. For the first time, the impact of carbonisate extracted from waste MDF on the tensile properties of the materials is presented. A scanning electron microscopy (SEM) study was carried out for a detailed analysis. The particle size of the carbonisations before and after comminution to a particle size of 0.5 mm was visualised. In addition, SEM studies of cross-sections of the composite samples after fracture were carried out to assess damage mechanisms and material structure after loading. Due to the limited number of studies on the recycling of MDF in carbonisate, these results highlight their importance for developing a closed-loop economy and indicate the need for further work on efficient methods for processing this type of waste.

To assess the effect of carbonisation on the structure and mechanical properties of composites, extensive and advanced testing methods are required. It was decided to use a static tensile test as the primary test for the newly produced composite material. The results obtained in this test were additionally decided to be analysed using metric entropy calculations, which make it possible to capture the significant qualitative changes occurring in the structure of the tested samples independent of the results of the static tensile test. In the context of epoxy composites with the addition of carbonisate, this analysis can contribute to a better understanding of the effect of this additive on the composite structure and its performance properties.

Studies carried out on isotropic [11,15] and anisotropic [16,17] materials confirm the suitability of using the $E_{K-S}$ calculation method to analyse the strength properties of various structural materials.

## 2. Materials and Methods

EM 1002/450/125 (by KROSGLASS, Krosno Poland) emulsion mat with random fibre direction was used as reinforcement for the composites with carbonisate. The mat used in the manufacturing process is characterised by a fibre diameter of 11 μm and a density of 450 g/m^2^. The matrix was epoxy resin Epidian^®^ 6 (by Ciech Sarzyna, Nowa Sarzyna, Poland) and hardener Z-1 (by Ciech Sarzyna, Nowa Sarzyna, Poland) was used, which is applied to the resin used. Table 1 summarises the properties of the epoxy resin Epidian^®^ 6.

**Figure 1:**
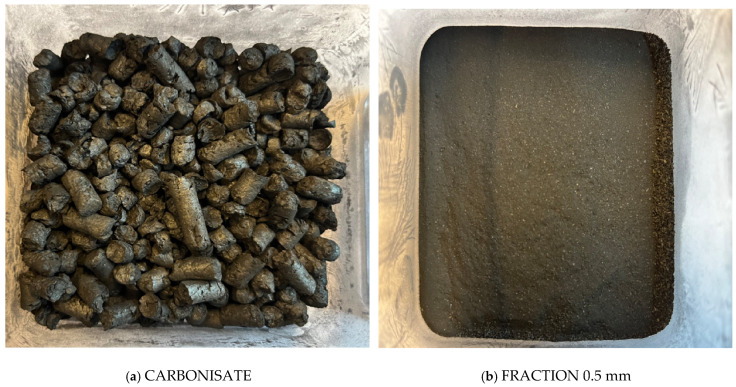
Carbonisate (**a**) after pyrolysis; (**b**) after crushing and screening to a 0.5 mm fraction.

Carbonisate formed by pyrolysis of furniture waste, including MDF, is characterised by irregular fraction (size range 7 mm to 1 mm) and well-developed porosity. However in order not to significantly deteriorate the mechanical properties of the composite, it is necessary to ensure the regular shape and optimal size of the additive particles. To this end, the carbonisate was finely crushed and then sieved using a sieve shaker to select particles of specific sizes, as shown in Figure 1.

The carbonisate, obtained by pyrolysis of MDF furniture boards, has a high carbon content of as much as 79.19% [18]. Other components include ash (6.95%) [19], nitrogen (4.43%) [18], hydrogen (2.99%) [18], chlorine (0.08%) [20], sulphur (0.07%) [20] and residues including oxides 6.29% [21]. The tests were commissioned from an external laboratory and carried out in accordance with the applicable standards, ensuring full compliance with the requirements.

**Figure 2:**
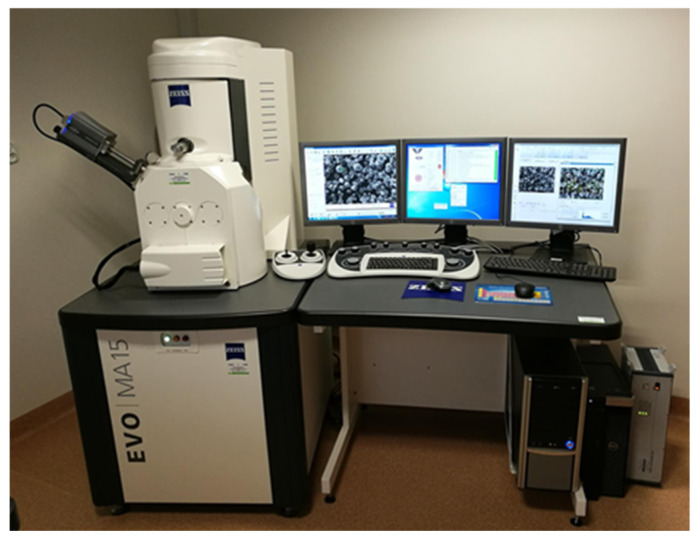
Zeiss EVO MA 15 scanning electron microscope.

To better understand the effect of the carbonisate particles on the properties of the composite, a Zeiss EVO MA 15 Figure 2 scanning electron microscope was used to study the microstructure of the carbonised and the composites made. The instrument allows electron images of samples to be obtained with a resolution of 3 nm at 30 kV. The range of possible magnifications is from 5 to 1,000,000 times. The microscope allows observation of samples weighing up to 500 g (with full mobility of the microscope table in XYZ directions) or up to 5 kg (then the movement of the table is limited to directions along the XY axis).

**Figure 3:**
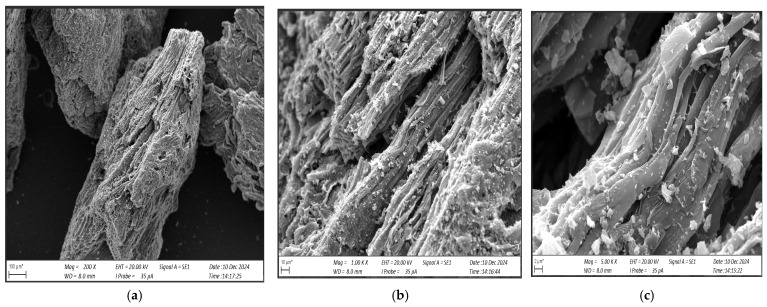
Carbonisate structure (**a**) magnification 200×; (**b**) magnification 1000×; (**c**) magnification 5000×.

**Figure 4:**
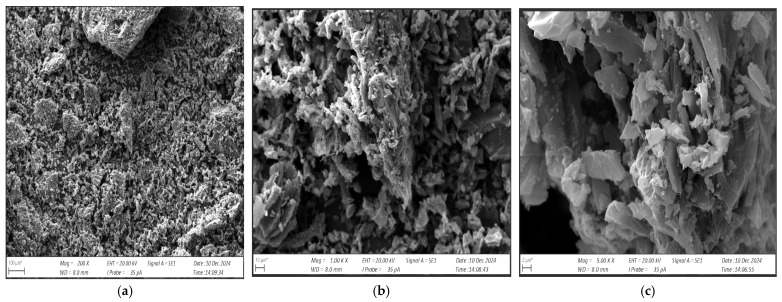
Structure of carbonisate with 0.5 mm fraction (**a**) magnification 200×; (**b**) magnification 1000×; (**c**) magnification 5000×.

Figure 3 shows an SEM image of the carbonisate obtained after pyrolysis, while Figure 4 shows carbonisate of 0.5 mm fraction.

The SEM images obtained showed that the carbonisate is characterised by high porosity, irregular particle shape and the presence of inclusions such as ash and residues of other components present in the carbonised.

The simplest method of forming composite elements is by hand lamination. It consists of placing reinforcement in a mould (successive layers of emulsion mat) and saturating its layers with Epidian^®^ 6 resin with hardener Z-1, after saturation the resin gels, cross-links and then cures. To make the composite with carbonisate, a mould had to be prepared using a distributor such as wax. The next step was to determine the proportions of resin, mat and carbonisate to saturate the entire mat and obtain the material with the best properties. Composite I and III, a material with no carbonisate added, was used as the base material against which the results obtained from variant II and IV composites were compared. Composites I and III consisted of 10 layers of emulsion matting infiltrated with Epidian^®^ 6 resin mixed in the specified proportion with hardener Z-1. In all the composites made, hardener was added in the amount of 13 g/100 g of matrix (Epidian^®^ 6 resin). The mass proportion of the emulsion matrix in material I was 40%, while in material III it was 35%, for materials with the addition of carbonisate for sample II 32.5% and for sample IV 30%, respectively. The carbonisate (by mass 7.5% and 5%) was mixed with Epidian^®^ 6 resin to obtain a material with comparable properties, and then Z-1 hardener was added.

To obtain a comparative result, samples without the addition of carbonisate and with the addition of carbonisate at the selected resin Epidian^®^ 6 content should be tested. The selected material variants are summarised in Table 2.

Based on the developed procedures for the proportion of individual components in composites by hand lamination, the test material was prepared. The test material consisted of plates corresponding to the size of the mould. The mould had dimensions of 900 × 300 × 10 mm (length × width × depth). Test specimens were prepared from the plates.

**Figure 5:**
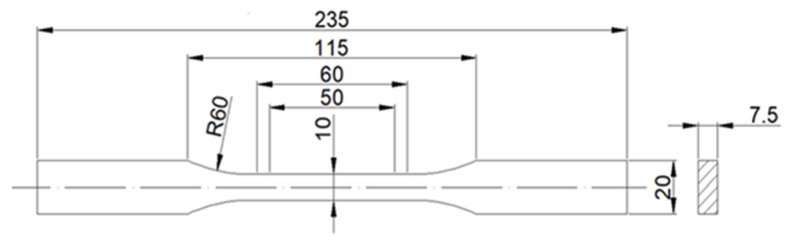
Shape and dimensions of samples for static tensile tests.

**Figure 6:**
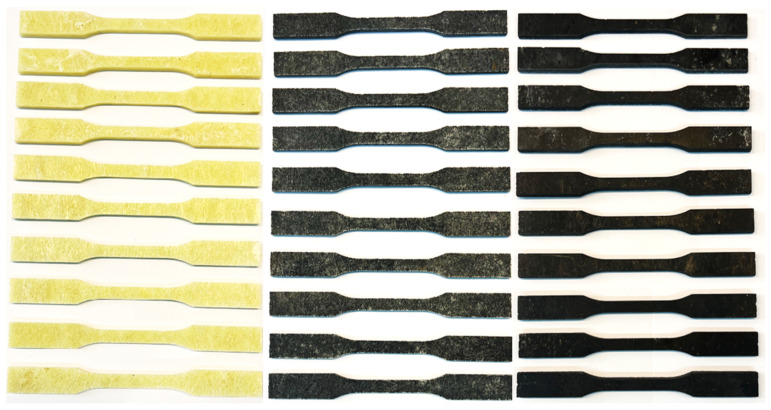
Samples of individual tested variants from the left: I, II and IV.

To determine the effect of the content of carbonisate on the properties of the produced composite materials, samples were prepared for static tensile testing by the requirements of the current standard [22], as shown in Figure 5. The composite material specimens were made by the water jet cutting method. This method ensured very accurate preservation of the made specimens, as well as avoiding the influence of temperature on changes in the structure of the material (in the case of mechanical processing). The prepared specimens for static tensile testing are shown in Figure 6.

## 3. Results

**Figure 7:**
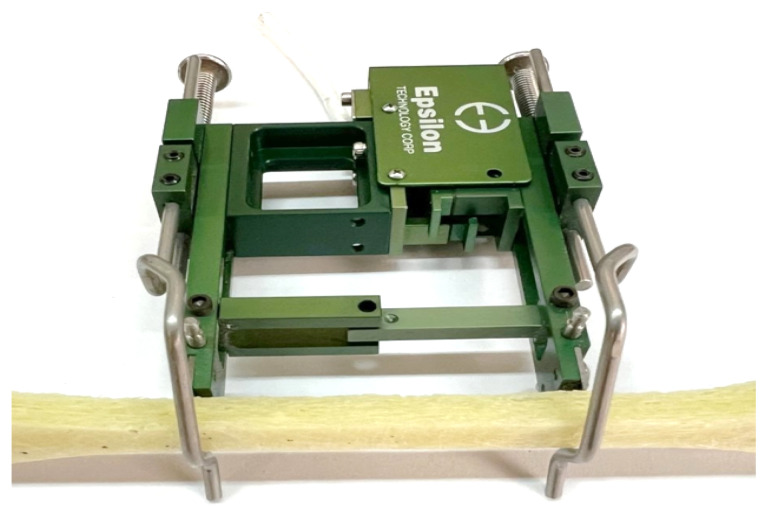
Variant I specimen prepared for static tensile test with Epsilon strain gauge type 3542.

Static tensile testing of the tested composites was carried out on a hydraulic-powered universal testing machine type MPMD P10B with TestXpert II software version 3.61. from Zwick and Roell. A strain gauge from Epsilon, type 3542, with an initial measurement length L_0_ of 50 mm, shown in Figure 7, was used to measure elongation during the test. 10 measurement tests were performed for each combination of specimens. The tensile speed during the static tensile test of all specimens was 1 mm/min. The average values of the static tensile test and strain of the tested composite materials are summarised in Table 3.

**Figure 8:**
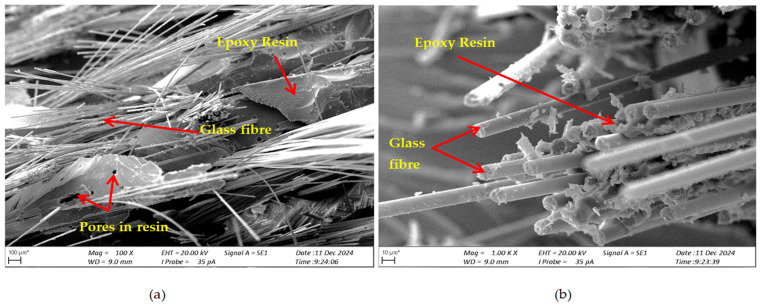
Structure of the composite (variant I) after static tensile test, cross-section after fracture (**a**) magnification 100×; (**b**) magnification 1000×.

**Figure 9:**
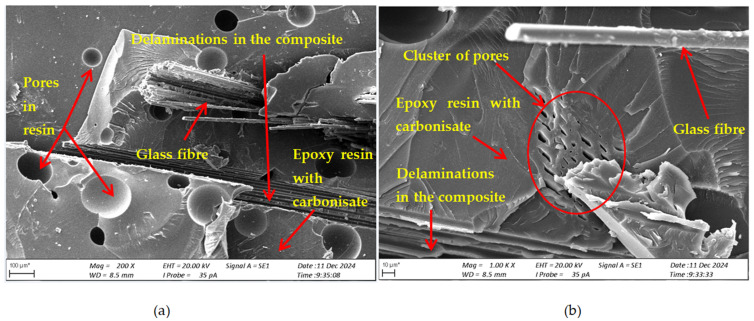
Structure of the composite (variant II) after static tensile test, cross-section after fracture (**a**) magnification 200×; (**b**) magnification 1000×.

**Figure 10:**
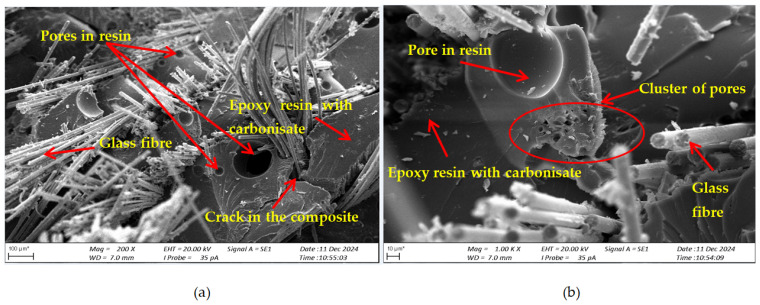
Structure of the composite (variant IV) after static tensile test, cross-section after fracture (**a**) magnification 200×; (**b**) magnification 1000×.

SEM studies of cross-sections of the composite specimens after fracture were performed to assess the damage mechanism and material structure after loading. Figure 8 shows the structure of the composite without the addition of carbonisate (variant I), Figure 9 shows the structure of the composite with the addition of carbonised for variant II and Figure 10 for variant IV.

Based on the obtained images of structures, in the analysed composites with the addition of carbonisate (variant II 7.5% and variant IV 5%), the occurrence of numerous pores, delaminations and cracks were noticed, which may indicate a number of potential problems related to their structure and production process, because manual lamination can significantly affect the structure and mechanical properties of composites with the addition of carbonisate. The main factors leading to these defects were, among others, the introduction of air during the manual lamination process. The presence of porosity, especially in excess, can lead to a decrease in the strength of the material and also affect its resistance to cracking. Delaminations and cracks within the composite suggest that there are problems with the adhesion of the resin to the carbonisate, which may result from the incorrect selection of the proportions of materials or the lack of appropriate chemical interaction between the components. Additionally, the quality of the carbonisate itself, especially if it comes from waste, maybe a factor influencing its effectiveness in the composite. The introduction of impurities such as ash or other component residues can negatively affect the structure of the material and lead to its weakening. In order to improve the mechanical and structural properties of the composite, it is necessary to optimise the proportions of resin and carbonisate.

**Figure 11:**
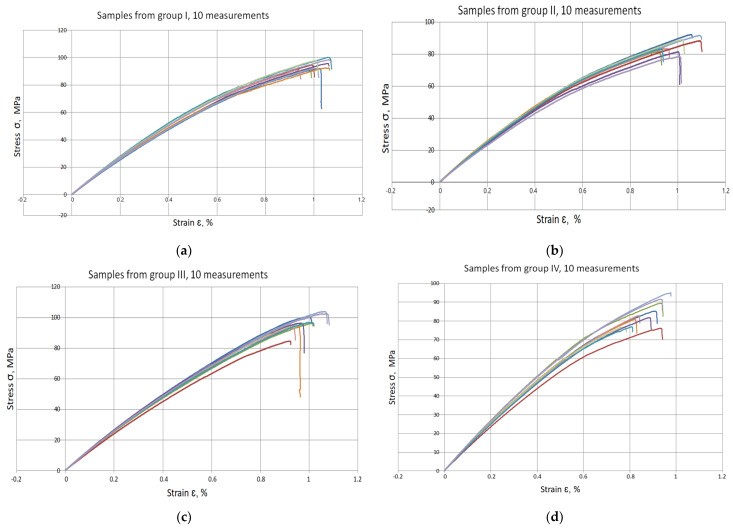
Tensile curves of composite materials (**a**) Group I specimens; (**b**) Group II specimens; (**c**) Group III specimens; (**d**) Group IV specimens. The lines in the graph represent the results of 10 measurements and the colours are used randomly.

Figure 11 shows the tensile curves obtained in the static tensile test of each of the variants of the tested material, I, II, III and IV, respectively.

From the data presented, one can see the effect of carbonisate on the mechanical properties of the materials in terms of stress (σ) and strain (ε). Comparison of samples without carbonisate (0% by weight). Samples I and III, which do not contain carbonisate, show stresses *σ* = 94.62 MPa and *σ* = 96.48 MPa, respectively. The deformations for these samples are ε = 1.017% and ε = 0.985%, respectively. These results indicate stable material properties in the initial state, with the differences in stress and strain mainly due to different resin/reinforcement ratios, suitable for variant I 60/40% and for variant III 65/35%. The effect of the addition of 0.5 mm carbonisate at 7.5% (by mass, specimen II) results in a reduction in R_m_ to σ = 86.07 MPa, a decrease in strength of about 9%. The deformation remains almost unchanged ε = 1.016%, suggesting that the addition of carbonisate does not significantly affect the ductility of the material. The effect of the addition of 0.5 mm carbonisate in an amount of 5% (by mass, samples IV) results in a reduction in R_m_ to σ = 83.66 MPa, which means a reduction in tensile strength of about 13% compared to variant III samples (without carbonisate). The deformation of the variant IV samples also decreased to a value of ε = 0.880%, a reduction of about 10.64% relative to the variant III samples.

The reduction in tensile strength and strain values with the introduction of carbonisate may indicate a weakening of the epoxy matrix cohesion due to the presence of the modifier, especially at lower weight fractions. A high carbonisate content of 7.5% has a less favourable effect on tension than a lower content of 5%, which may suggest that the optimal carbonisate content is in the ≤5% range.

### 3.1. Method of Calculation $E_{K-S}$

**Figure 12:**
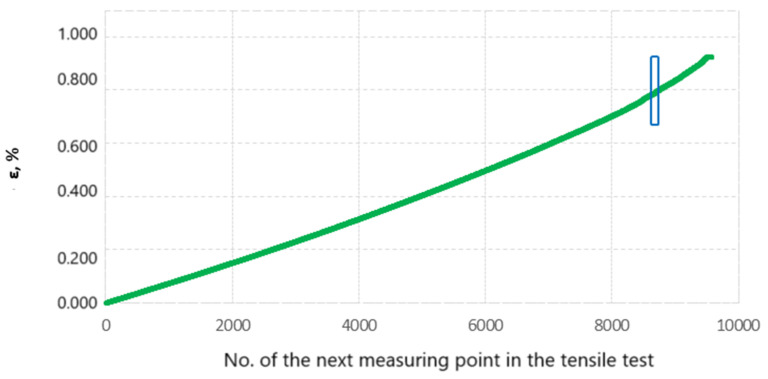
The set of elongation values ε and the $E_{K-S}$ calculated for them.

The Kolgomorov–Sinai $E_{K-S}$ metric entropy calculation was performed on sets of relative elongation values ε obtained during static tensile testing of all specimens from groups I, II, III and IV. To explain the $E_{K-S}$ calculation method, we will use an example of calculations made for the specimen designated III-2. During the static tensile test of specimen No. III-2, 9575 measuring points were recorded. Figure 12 shows a set of values of relative elongation, percentage ε as a function of successive measurement points.

The calculation of $E_{K-S}$ entropy involves dividing a 9575-point set of ε values into intervals. In this case, the intervals contained 40 consecutive values of ε, which corresponded to 40 successive elongation values recorded. The first interval of the set of values starts at the first measurement point and ends at the fortieth measurement point. the next interval starts at the second measurement point and ends at the forty-first and so on. [15]. Each 40-number interval was further divided into four subintervals. The value of the metric entropy was counted for each interval according to the principle defined by Kolmogorov–Sinai [23]:

(1) $$E_{K-S}=-\sum_{i=1}^{N}p_{i}\ln p_{i}.$$

where:

*p*—probability, *i*—of this state.

**Figure 13:**
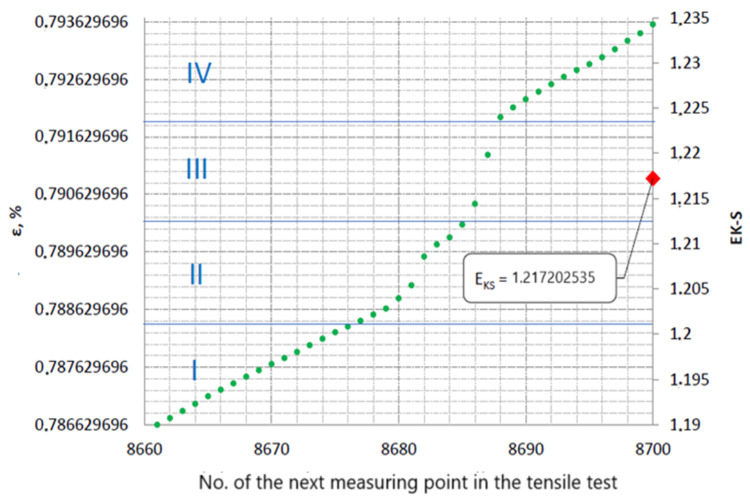
Excerpt from the tensile curve of specimen No. III-2, containing one 40-number interval of successively recorded values of relative elongation ε and marked IV subintervals of this interval.

To explain the $E_{K-S}$ counting process, Figure 13 shows one selected interval of 40 numbers, which comes from the set of elongation values recorded during the tensile test of specimen No. III-2. This interval is highlighted in Figure 12 with a blue rectangle. The selected interval was between 8660 and 8700 measurement points. In Figure 13, the values of the percentage relative elongation ε contained in the aforementioned interval are shown in green.

The lowest value of elongation ε recorded in this range was 0.786629696%, while the highest was 0.793583362%. The interval was divided into four equal subintervals in terms of relative elongation values (0.790106529% each), marked in the figure with Roman numerals from I to IV and separated by horizontal blue lines.

Table 4 shows the detailed calculation of $E_{K-S}$ Entropy for the selected interval shown in Figure 13. The probability *p_i_* that an element of the adopted interval belongs to the corresponding subinterval (from I to IV) was calculated. This made it possible to calculate the $E_{K-S}$ entropy for the selected 40-number interval according to Formula (1). The $E_{K-S}$ entropy value of 1.217202535 was assigned to the last point of the calculated interval:

$$E_{K-S}=-\sum_{i=1}^{N}p_{i}\ln p_{i}=-\left(-0.366516293-0.335622347-0.149786614-0.365277281\right)=1.217202535$$

**Table 4:** Detailed calculation of $E_{K-S}$ entropy for the selected 40-number interval shown in Figure 13.

	4 Subdivisions Adopted			
	I	II	III	IV
	min range 0.786629696	min range 0. 0.788368113	min range 0. 0.790106529	min range 0. 0.791844946
40 number range adopted	0.786629696	0.788433487	0.790466041	0.791971933
	0.786746396	0.788542098	0.791315734	0.792138571
	0.786867303	0.788644400		0.792279971
	0.786992201	0.788824952		0.792413659
	0.787123354	0.789048214		0.792542763
	0.787237143	0.789546403		0.792670950
	0.787347211	0.789757101		0.792788083
	0.787462617	0.789879572		0.792891247
	0.787576298	0.790104453		0.793010536
	0.787683237			0.793150426
	0.787784730			0.793299376
	0.787892780			0.793427833
	0.788009773			0.793583362
	0.788121458			
	0.788235031			
	0.788337063			
	max range <0.788368113	max range <0.790106529	max range <0.791844946	max range 0.793583362
$p_{i}$	0.400	0.225	0.050	0.325
$\ln p_{i}$	−0.916290732	−1.491654877	−2.995732274	−1.123930097
$p_{i}\ln p_{i}$	−0.366516293	−0.335622347	−0.149786614	−0.365277281
$E_{K-S}$	1.217202535			

To calculate $E_{K-S}$ for the entire set of elongation values ε recorded during the test, it is necessary to perform the above calculations on all intervals from the set of values [17]. The number of measurement points recorded during the static tensile test was 9575 points. The calculation of metric entropy was therefore performed for 9535 intervals of 40 points of this using Equation (1).

**Figure 14:**
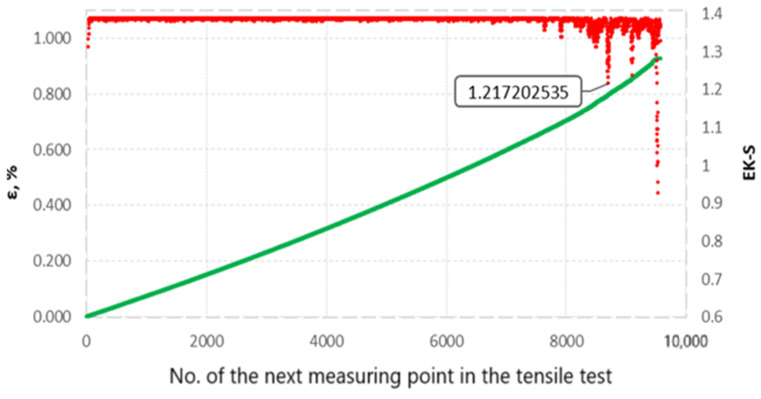
The set of elongation ε (green) and the calculated $E_{K-S}$ values for each of the 9535 intervals (red), compiled in a single figure. The value calculated for an entropy interval was assigned to the last point of that interval.

The “Entropy K–S” software version 1.20 (Authors: Łukasz Mrugała—programmer, code author; Grzegorz Hajdukiewicz—functionality concept; Gdańsk, Poland 2021) was used for the calculations, which enabled calculation of metric entropy for each interval and obtaining a set of metric entropy values. The metric entropy values are presented graphically and compared with the elongation plot ε = f(t) in Figure 14.

### 3.2. Analysis of Strength Properties Using Metric Entropy Calculations

**Figure 15:**
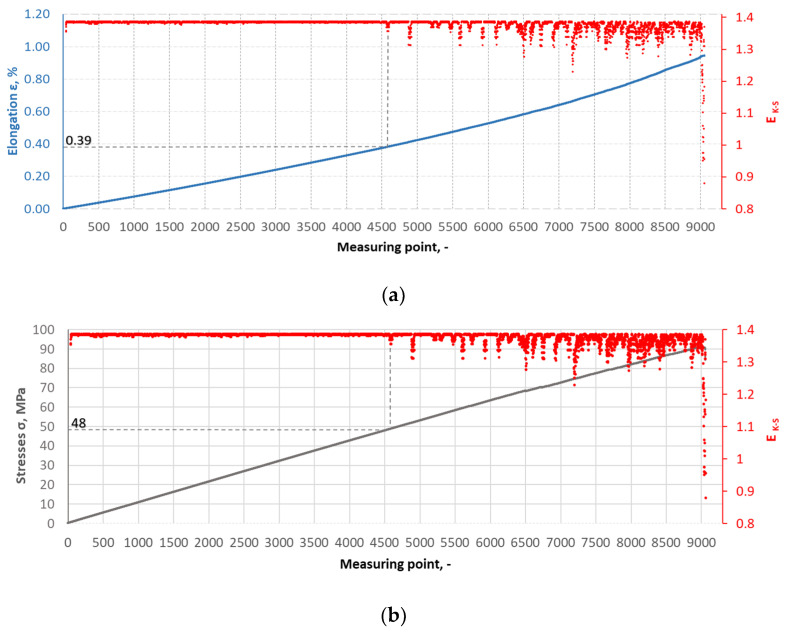
(**a**) The course of the strain curve for the I.8 composite sample; (**b**) the results of the metric entropy calculations $E_{K-S}$ as a function of the test time for the I.8 composite sample.

**Figure 16:**
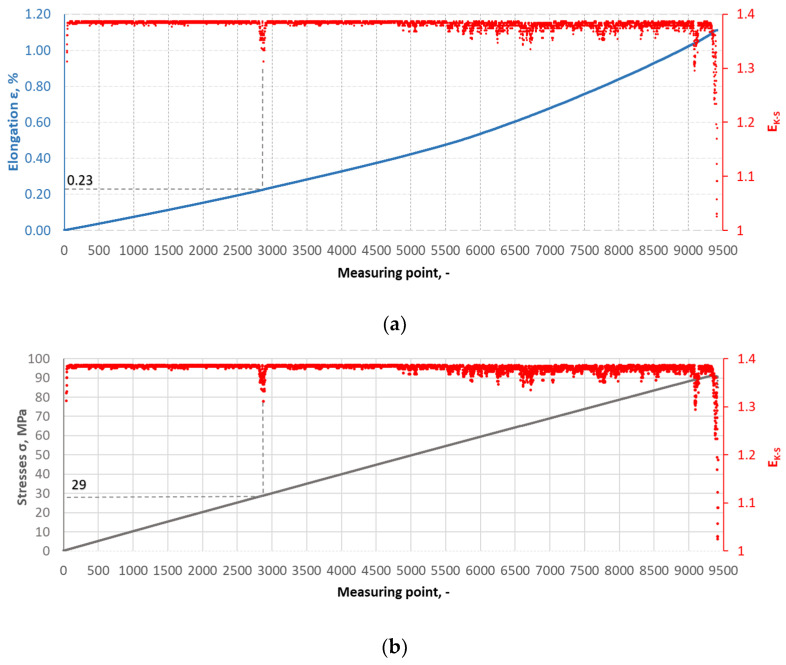
(**a**) The course of the strain curve for the II.6 composite sample; (**b**) the results of the metric entropy calculations $E_{K-S}$ as a function of the test time for the II.6 composite sample.

**Figure 17:**
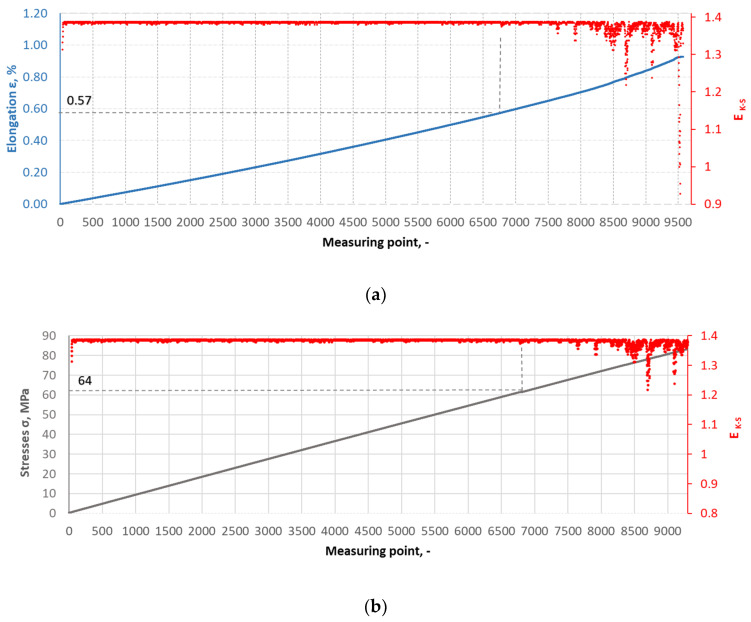
(**a**) The course of the strain curve for the III.2 composite sample; (**b**) the results of the metric entropy calculations $E_{K-S}$ as a function of the test time for the III.2 composite sample.

**Figure 18:**
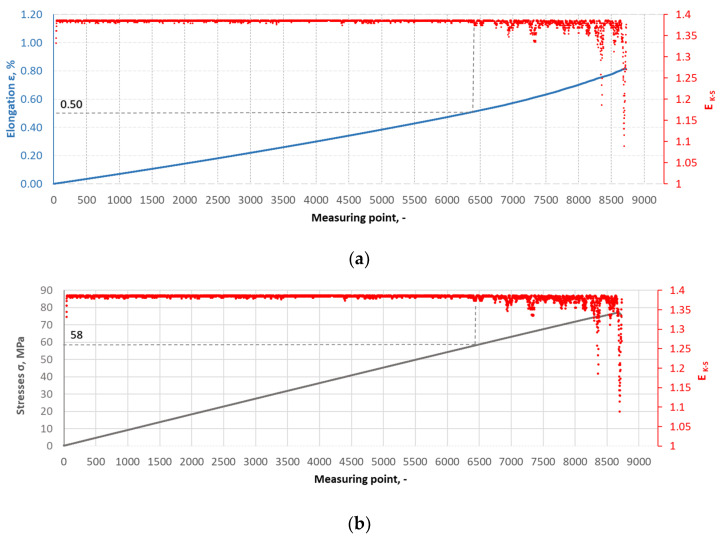
(**a**) The course of the strain curve for the IV.5 composite sample; (**b**) the results of the metric entropy calculations $E_{K-S}$ as a function of the test time for the IV.5 composite sample.

The Kolmogorov–Synai metric entropy method was used to analyse the degree of homogeneity of the structure of epoxy composites with the addition of carbonisate. This method enabled the development of model entropy profiles for materials with different carbonisate contents (5% and 7.5%). Figure 15, Figure 16, Figure 17 and Figure 18 show selected tensile curves that represent each variant of the tested material.

For each analysed composite material variant, a characteristic point was identified at which the value of the metric entropy $E_{K-S}$ begins to decrease. The decrease in this value reflects a change in the dynamics of the processes taking place. At this point, the composite sample shows a dynamic response to a uniformly increasing tensile force, which indicates critical qualitative changes inside the material. These changes may be related to phenomena such as the initiation of cracks in the matrix, separation of composite fibres or delamination resulting from the presence of carbonisate. After determining the measuring point at which the metric entropy $E_{K-S}$ decreases, the strain value ${ԑ}_{K-S}$ assigned to this point is read.

Table 5 compares the average values of σ and ε obtained in the static tensile test and the average values of relative strain ${ԑ}_{K-S}$ and the corresponding stress values $\sigma_{K-S}$ obtained using Kolgomorov–Sinai metric entropy calculations $E_{K-S}$.

## 4. Discussion

In the conducted studies on the effect of carbonisate on the mechanical properties of epoxy composites, a clear effect of this additive on tensile strength, and elongation was observed. These results indicate a compromise between the benefits associated with the use of carbonisate and the potential weakening of the mechanical integrity of materials.

For the variant I sample without carbonisate addition, the tensile strength was obtained at the level of σ = 94.62 MPa and elongation ԑ = 1.017%. The addition of 7.5% of carbonisate (variant II samples) with a fraction of 0.5 mm resulted in a reduction of the tensile strength to σ = 86.07 MPa, with almost unchanged elongation ԑ = 1.016%. A similar trend was observed in sample III, where the strength dropped from σ = 96.48 MPa to σ = 83.66 MPa (variant IV samples) after the addition of 5% of carbonisate, with a simultaneous reduction of elongation from ԑ = 0.985% to ԑ = 0.880%. These results suggest that the addition of carbonisate weakens the bond between reinforcement fibres and resin. The results of the calculations of the entropy metric also showed a decrease for the samples with carbonisate. For the composites of variant I without the addition of carbonisate, the entropy value was $\sigma_{K-S}$ = 48 MPa and elongation ${ԑ}_{K-S}$ = 0.39%, while for the sample with carbonisate, variant II, these values decreased to $\sigma_{K-S}$= 27 MPa and ${ԑ}_{K-S}$ = 0.22%. Similarly, in the case of composites of variant III, the entropy decreased from $\sigma_{K-S}$= 64 MPa (elongation ${ԑ}_{K-S}$ = 0.56%) to $\sigma_{K-S}$= 58 MPa for sample IV (elongation ${ԑ}_{K-S}$ = 0.50%). The decrease in these parameters indicates a more ordered but less flexible structure, which may be a consequence of introducing a filler with relatively large particles (0.5 mm). The decrease in the tensile strength and plasticity of the material may result from the decreased adhesion between the reinforcement and the composite matrix caused by the presence of the additive. This is confirmed by the results of SEM structure analysis carried out for variants II and IV containing carbonisate, showing the presence of cracks, delaminations and numerous pores.

## 5. Conclusions

The conducted studies have shown that the addition of 5% and 7.5% (by weight) of carbonisate significantly affects the mechanical properties of epoxy composites, causing a decrease in the tensile strength R_m_. At the same time, they have shown that a higher, i.e., 7.5% percentage addition of carbonisate has a significant effect on the plasticity of the material. Adding carbonisate resulted in a decrease in mechanical strength, which can be explained by the disruption of adhesive connections between the fibres and the composite matrix (the formation of structural defects at the phase boundary). Analyses performed using scanning electron microscopy (SEM) revealed the presence of cracks, pores and delaminations, which confirm this hypothesis. The use of metric entropy calculations to illustrate the change in the dynamics of deformation of the tested samples has shown significant qualitative changes occurring inside the materials at stress values σ*_K-S_* and strains ԑ*_K-S_* lower than the obtained results of the static tensile test, i.e., σ and ԑ. This should be understood as meaning that the actual ability of the composite (pure and with the addition of carbonisate) to absorb and dissipate energy during tensile loads is lower than the results of the static tensile test. Analysing the results of the $E_{K-S}$ tests and calculations, it was noted that a higher share of fibres in the composite composition (ratio 65/35) led to higher tensile strength results compared to samples with a lower share of fibres (60/40), which emphasizes the important role of the proportions of components in shaping the mechanical properties of composites. Optimisation of the amount and size of carbonisate particles, as well as reducing its negative effect on the adhesive bonds of fibres with the matrix, may be crucial for obtaining better mechanical properties of the composites in question. The final selection of the material composition should be ultimately adapted to the specific application requirements and target properties.

## Figures and Tables

**Table 1:** Properties of the epoxy resin Epidian^®^ 6.

Parameter	Unit	Value
Viscosity at 25 °C	mPa s	1000–1500
Density at 20 °C	g/cm^3^	1.17
Gelation time, at 25 °C	min	33
Flash point	°C	>200
Epoxy number	mol/100 g	0.510–0.540

**Table 2:** Content of individual composite materials made by hand lamination.

No.	Fraction Carbonisate [mm]	Number of Layers Mats	Content Resin % (by Mass)	Content Mats % (by Mass)	Content Carbonisate % (by Mass)	Determination Samples
1	-	10	60	40	0	I
2	0.5	10	60	32.5	7.5	II
3	-	10	65	35	0	III
4	0.5	10	65	30	5	IV

**Table 3:** Average values of static tensile test and strain for samples with different percentages of carbonisation and fractions.

No.	Fraction Carbonisate [mm]	Content Carbonisate % (by Mass)	Determination Samples	σ Stress MPa	Difference Percentage %	ԑ % Strain	Difference Percentage %
1	-	0	I	94.62	9.032	1.017	0.098
2	0.5	7.5	II	86.07		1.016	
3	-	0	III	96.48	13.29	0.985	10.64
4	0.5	5	IV	83.66		0.880	

**Table 5:** Average values of strength parameters obtained in the static tensile test and average results obtained by metric entropy calculations.

Determination Samples	ԑ, %	σ, MPa	${ԑ}_{K-S}$, %	Error Bar ${ԑ}_{K-S}$, %	$\sigma_{K-S}$, MPa	Error Bar $\sigma_{K-S}$, MPa	$\mathrm{Changing}\;\mathrm{the}\;{ԑ}_{K-S}$ Relative to *ε*, %	$\mathrm{Change}\;\mathrm{in}\;\sigma_{K-S}$ Relative to *σ*, %
I	1.017	94.62	0.39	0.30–0.48	48	38–58	62	49
II	1.016	86.07	0.22	0.18–0.29	27	22–35	78	69
III	0.985	96.48	0.56	0.46–0.60	64	53–70	43	34
IV	0.880	83.66	0.50	0.44–0.53	58	54–60	43	31

## Data Availability

The original contributions presented in this study are included in the article. Further inquiries can be directed to the corresponding author.
